# ^68^Ga-FAPI-PET/CT in extra-cervical CUP: Head-to-Head Comparison of ^68^Ga-FAPI-46 with ^18^F-FDG in 13 patients

**DOI:** 10.1007/s00259-026-07916-0

**Published:** 2026-05-22

**Authors:** Anna-Maria Spektor, Sergio Armando Zapata Bonilla, Philipp Mildenberger, Anton Kilburg, Daniela Driess, Erik Winter, Michael Kloth, Mathias Schreckenberger, Tobias Bäuerle, Thomas Kindler, Uwe Haberkorn, Maria Pouyiourou, Alwin Krämer, Tilmann Bochtler, Manuel Röhrich

**Affiliations:** 1 Department of Nuclear Medicine, University Medical Center, Johannes Gutenberg University Mainz, Langenbeckstraße 1, 55131 Mainz, Germany, Germany; 2 University Cancer Center, University Medical Center, Johannes Gutenberg University, Langenbeckstr. 1, 55131 Mainz, Germany; 3 Institute of Medical Biometry, Epidemiology and Informatics, Johannes Gutenberg University Mainz, Mainz, Germany; 4 Department of Diagnostic and Interventional Radiology, University Medical Center, Johannes Gutenberg-University Mainz, Mainz, Germany; 5 Department of Nuclear Medicine, University Hospital Heidelberg, Heidelberg, Germany; 6 Department of Pathology, University Medical Center, Johannes Gutenberg-University Mainz, Mainz, Germany; 7 3Rd Medical Department, University Medical Center, Johannes Gutenberg University Mainz, Mainz, Germany; 8 TRON-Translational Oncology, University Medical Center, Johannes Gutenberg University Mainz, Mainz, Germany; 9 German Cancer Consortium (DKTK), Partner Site Frankfurt/Mainz, a partnership between DKFZ and University Medical Center Mainz, Mainz, Germany; 10 Translational Lung Research Center Heidelberg (TLRC), Member of the German Center for Lung Research DZL, Heidelberg, Germany; 11 Clinical Cooperation Unit Nuclear Medicine, German Cancer Research Center (DKFZ), Heidelberg, Germany; 12 Clinical Cooperation Unit Molecular Hematology/Oncology, German Cancer Research Center (DKFZ) and Department of Internal Medicine V, University of Heidelberg, Heidelberg, Germany; 13 Department of Internal Medicine V, University of Heidelberg, Heidelberg, Germany; 14 Department of Medical Oncology, Medical Faculty Heidelberg, Heidelberg University Hospital, Heidelberg, Germany

**Keywords:** CUP, FAPI, FDG, Extra-cervical, Single-site, Oligometastatic

## Abstract

**Purpose:**

Oncological treatment in a substantial portion of patients with cancer of unknown primary (CUP) remains challenging due to limitations of conventional imaging and positron emission tomography/computed tomography with ^18^Fluor-fluorodeoxyglucose (^18^F-FDG-PET/CT). In head and neck-like CUP (HNCUP), several studies found significantly higher tracer-uptake and detection rates of primary tumors in ^68^Gallium-labeled fibroblast activation protein inhibitor-PET/CT (^68^Ga-FAPI-PET/CT). Here, we address a gap in CUP literature by retrospectively evaluating the diagnostic accuracy of both tracer in a head-to-head comparison of patients with single-site and oligometastatic extra-cervical CUP.

**Methods:**

13 patients with extra-cervical CUP underwent both ^18^F-FDG- and ^68^Ga-FAPI-PET/CT. Suspicious PET-positive lesions were delineated using the volume of interest-technique (50%-isocontour) followed by analysis of maximum and mean standardized uptake values (SUVmax/mean), target-to-background ratios (TBRmax/mean) and tumor-to-tissue ratios (TTRmax/mean). Descriptive analysis was performed and log-transformation was applied to meet model assumptions and stabilize variance. The difference between log-transformed ^68^Ga-FAPI- and ^18^F-FDG-uptake values was used as outcome variables.

**Results:**

21 metastases of 3 patients with single-site and 6 patients with oligometastasized CUP were analyzed. 4 patients with previously extirpated metastases showed no uptake. No increased detection rate of primary tumors could be observed by either tracer. Differences of TBRmax in all metastatic sites and of TTRmax in organ metastases were significant. ^68^Ga-FAPI-PET/CT lead to detection of 5/21 additional metastases which were not clearly distinguishable in ^18^F-FDG-PET/CT.

**Conclusion:**

Due to higher tissue contrast in organs, our findings suggest that ^68^Ga-FAPI-PET/CT appears favourable compared to ^18^F-FDG-PET/CT particularly in patients with suspected distant metastases other than lymph nodes.

**Supplementary Information:**

The online version contains supplementary material available at 10.1007/s00259-026-07916-0.

## Introduction

Cancer of unknown primary (CUP) remains an often frustrating diagnostic and therapeutic challenge in daily oncological practice. Despite significant advances in immunohistochemistry, molecular profiling and imaging modalities, a substantial portion of patients still presents with metastatic disease without an identifiable primary tumor [1–4]. Partly for this reason median overall survival remains under 12 months in most reported series [5–7]. The high mortality rate in CUP is compounded by the absence of a truly standardized systemic therapy. While empiric chemotherapy, typically platinum-based, remains common, outcomes are heterogeneous and defined prognostically favorable sub-groups are small, accounting for only about 15–20% of patients [8].

In this context, improving diagnostic accuracy is critically important. A rigorous clinical and histological work up is of essence, though in some cases it could lead to delays in the implementation of a definitive therapy. A key component of the diagnostic pathway is imaging, with either computed tomography (CT) or magnetic resonance imaging (MRI) of neck, thorax, abdomen and pelvis being mandatory on the standardized workflow for the identification of a primary tumor and additional metastatic manifestations as per current guidelines [1]. Furthermore, functional imaging plays a growing role and is recommended by guidelines for patients with single-site and oligometastatic as well as head and neck-like CUP (HNCUP) subtypes. The conventional standard is positron emission tomography combined with CT with ^18^Fluor-fluorodeoxyglucose (^18^F-FDG-PET/CT), which visualizes increased glucose metabolism as a surrogate of malignant activity. Yet the modality has known limitations: uptake is variable among tumor types, false-positive findings may occur from inflammatory processes, and the detection rate of primary tumors in CUP remains modest with meta‐analyses suggesting a primary tumor detection rate of around 30–40% for ^18^F-FDG-PET/CT in CUP [9, 10].

Recently, interest has grown in a novel tracer class targeting the fibroblast activation protein (FAP), which is enriched in cancer-associated fibroblasts and tumor stroma of many epithelial malignancies [11, 12]. Radiolabeled FAP inhibitors (FAPI) represent a promising alternative to FDG, offering potentially improved tumor-to-background contrast, uptake in lesions with low glycolytic activity, and reduced false-positive findings. Several studies across diverse tumor types report higher uptake, higher target-to-background ratios and improved lesion detection with ⁶⁸Ga- or ^18^F-labelled-FAPI-tracers compared to conventional ^18^F-FDG-PET/CT, in other challenging entities, like pancreatic adenocarcinoma [13, 14].

In the specific setting of HNCUP, early reports of ⁶⁸Ga-FAPI-PET/CT are encouraging: several studies found significantly higher detection rates of the primary tumor and higher tracer-uptake in FAPI-imaging [15–17]. Another upside of FAPI-imaging is that it may also offer a potential therapeutic target in the context of a theragnostic approach. Radioligand therapy targeting FAP-expressing tumors is under investigation and could represent a novel treatment avenue in the future, although this remains investigational and is not yet validated across entities, nor in the context of CUP [18–20]. To date, there have been only a small number of studies comparing ^68^Ga-FAPI- versus ^18^F-FDG-PET/CT in extra-cervical CUP [17, 21]. Here, we address this gap in current CUP research by retrospectively evaluating the diagnostic accuracy of ⁶⁸Ga-FAPI- and ^18^F-FDG-PET/CT in a head-to-head comparison of patients with single-site and oligometastatic extra-cervical CUP.

## Methods

### Patient characteristics

13 patients with extra-cervical CUP underwent both ^18^F-FDG- and ^68^Ga-FAPI-PET/CT as part of an optimized staging protocol. 3 patients were examined at the University Medical Center Mainz, Germany and 10 patients at the University Hospital Heidelberg, Germany. All patients from both sites gave written informed consent to undergo ^18^F-FDG-PET/CT and ^68^Ga-FAPI-PET/CT according to national regulations, the Declaration of Helsinki and Good Clinical Practice (GCP). Retrospective analysis of imaging, clinical, and pathologic data was approved by the local institutional review board (study number S-115/2020).

### PET/CT-Imaging

^18^F-FDG PET/CT was performed according to standard care as previously described [22]. Synthesis and labeling of ^68^Ga-FAPI-46 was conducted as previously reported [23–25]. For PET imaging, a Siemens Biograph mCT Flow scanner was used, according to previously published protocols [26]. Shortly, after a low-dose CT without contrast, 3-dimensional PET-scans were acquired (matrix, 200 × 200), reconstructions performed, and emission data corrected for attenuation. For all patients, PET-scans were acquired 60 min (min.) post injection (p. i.) of ^68^Ga-FAPI-46 and ^18^F-FDG.

### Analysis of PET data

All suspicious PET-positive lesions were delineated using the volume of interest (VOI) technique (50% isocontour) followed by lesion-wise analysis of maximum and mean standardized uptake values (SUVmax/mean), target-to-background ratios (TBR) (SUVmean of blood pool, defined by a VOI with a diameter of 10 mm in the descending aorta immediately after the aortic arch) and tumor-to-tissue ratio (TTR) (SUVmean of surrounding tissue, e.g. liver, bone, soft-tissue) to address the patient-specific effect. Lesions were classified based on radiological characteristics, clinical evolution, and, when available, histopathological confirmation. Visual detectability of the lesions was assessed independently by two nuclear medicine physicians (AS, MR). All lesions suspected in PET/CT were classified by a radiologist (AK) according to their CT-morphology and, where applicable, compared with further imaging (contrast enhances (CE)-CT/CE-MRI) and imaging follow-up.

### Statistical analysis

Statistical analysis was performed descriptively for patients’ characteristics, SUV, TBR and TTR using Graph Pad Prism 10.2.3. To address the paired nature of the data, we used the difference between log-transformed FAPI- and FDG-uptake values as the outcome variable. This approach is equivalent to modeling the log-ratio (log(FAPI/FDG)), allowing us to report back-transformed results as geometric mean ratios (FAPI/FDG) for improved interpretability. Given the skewed distribution of the uptake values, log-transformation was applied to meet model assumptions and stabilize variance. Due to the small sample size, which precluded reliable Wald-type inference, we employed a wild bootstrap resampling procedure with 1000 iterations to construct 95% confidence intervals for the fixed effects. The wild bootstrap with an auxiliary distance as introduced by Webb (2023) was chosen to best accommodate the small sample and the clustered nature of the data [27]. We further used this bootstrap method to derive p-values. Given the exploratory and hence hypothesis-generating nature of this study, a major focus of the analysis was on unadjusted p-values. For context, and to control the family-wise error rate, we also report p-values adjusted using the Bonferroni-Holm method. We interpreted non-overlap of the 95% interval with 1 as indicating statistical significance at the 5% level. Given the exploratory nature of this study, no adjustment for the multiple testing situation was made.

## Results

### Patient characteristics

In the final analysis, 9 women and 4 men were included. The mean age at imaging time point was 68.5 ± 6.95 years. Baseline clinical characteristics of these 13 patients are presented in Table 1. At the time point of CUP diagnosis, 6 patients presented as single-site CUP and 7 patients as oligometastatic CUP. Prior to PET/CT imaging, 2 patients were treatment-naïve, 5 patients underwent only resection of metastases, 3 patients had a resection of metastases and systemic therapy, 3 patients had received only systemic therapy. The most common systemic therapy was chemotherapy (5/6 patients); a smaller proportion additionally received immune checkpoint inhibitors (3/5 patients). One patient received only immune checkpoint inhibitors prior PET/CT. Histopathological reports were available for all patients, including detailed immunohistochemistry (IHC). Adenocarcinoma (46.2%) was the most common histological subtype, followed by squamous cell carcinoma (38.5%), urothelial and sarcomatoid carcinoma (7.7%, respectively). Molecular profiling via next-generation sequencing (NGS) was performed in 7 patients. Additional molecular and immunohistochemical (IHC) details are provided in supplemental Table 1. At imaging time point, metastases were detected in 9/13 patients. The most common metastatic site were the lymph nodes, followed by bones, liver, pleura and brain. Three patients had single-site CUP, six patients oligometastatic CUP and four patients showed no further metastases after removal of the initial metastasis. The median interval between ^18^F-FDG- and ⁶⁸Ga-FAPI-PET/CT was 6 days (1–26 days); while 11 patients had an interval between both examinations under 10 days, 2 patients were examined by ^68^Ga-FAPI-PET/CT 26 days after ^18^F-FDG-PET/CT. Most patients underwent ^18^F-FDG-PET/CT imaging first (92.3%). Patient characteristics at imaging time point are shown in Table 2.

**Table 1:** Demographics and clinical patient characteristics

Characteristics	Data (patient number: 13)
*Demographics*	
Mean age ± SD	68.5 ± 6.95
Female	9 (69.2%)
Male	4 (30.8%)
*Localization of metastases at initial diagnosis*	
Single-site CUP	6 (46.2%)
Oligometastatic CUP	7 (53.8%)
Lymph nodes	7 (53.8%)
Bones	3 (23.1%)
Liver	2 (15.4%)
Pleura	2 (15.4%)
Peritoneum	2 (15.4%)
Brain	1 (7.7%)
Pituitary gland	1 (7.7%)
Rectus abdominis muscle	1 (7.7%)
Abdominal wall	1 (7.7%)
*Histological subtype*	
Histology report available	13 (100%)
Adenocarcinoma	6 (46.2%)
Squamous epithelial	5 (38.5%)
Sarcomatoid	1 (7.7%)
Urothelial	1 (7.7%)
Immunohistochemical profile available	13 (100%)
NGS available	7 (53.8%)
Previous therapies*	
Resection of metastases	8 (61.5%)
Any systemic therapy previous to imaging	6 (46.2%)
Previous Chemotherapy	5 (38.5%)
Previous targeted immunotherapy	4 (30.8%)
Abbreviations: * Treated patients may have presented with more than one therapy line; SD: standard deviation; NGS: next-generation sequencing

**Table 2:** Patient characteristics at imaging time point

Characteristics	Data (patient number: 13)		
PET/CT-Imaging			
Median time interval between ^18^F-FDG- and ^68^Ga-FAPI-PET/CT (range)	6 days (1–26 days)		
^18^F-FDG-PET/CT first	12 (92.3%)		
^68^Ga-FAPI-PET/CT first	1 (7.7%)		
Metastases at imaging time point			
Number of patients with no metastases after resection of initial metastasis	4 (30.8%)		
Number of patients with metastases after resection of initial metastasis	9 (69.2%)		
Single-site CUP	3 (33.3%)		
Oligometastatic CUP	6 (66.7%)		
Localization of metastases at imaging time point and detection rate			
Metastatic sites	N	Detected by FAPI	Detected by FDG
Lymph nodes	9	9 (100%)	9 (100%)
Bones	4	4 (100%)	2 (50%)
Liver	3	3 (100%)	1 (33.3%)
Lung	2	2 (100%)	2 (100%)
Pleura	1	1 (100%)	1 (100%)
Peritoneum	1	1 (100%)	0
Spleen	1	1 (100%)	0

Abbreviations: N: number; ^18^F-FDG: ^18^Fluor-labeled fluordeoxyglucose; ^68^Ga-FAPI: ^68^Gallium-labeled fibroblast activation protein inhibitor; PET/CT: positron emission tomography with computed tomography;

### Tracer-uptake in all metastatic sites

**Fig. 1:**
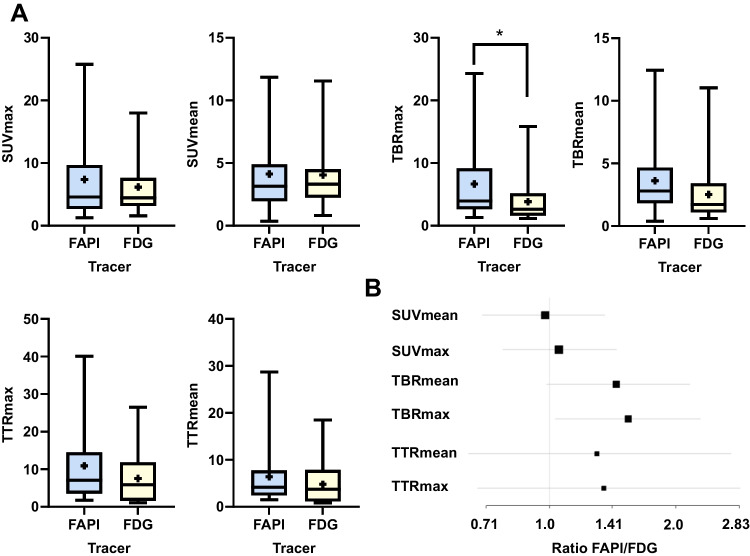
**A** Box-Whisker-Plots of maximum and mean standardized uptake values (SUVmax/mean), tumor-to-background ratios (TBRmax/mean) and tumor-to-tissue ratios (TTRmax/mean) of all metastatic lesions (9 lymph nodes, 4 bone, 3 liver, 1 peritoneal carcinomatosis bordering the liver dome, 2 lung, 1 pleura carcinomatosis, 1 spleen) of 9 patients with extra-cervical CUP who were examined with positron emission tomography combined with computed tomography (PET/CT) with ^68^Gallium-labelled fibroblast activation protein inhibitor (^68^Ga-FAPI) and ^18^Fluor-labelled fluorodeoxyglucose (^18^F-FDG). Significant differences (*) were only observed for TBRmax. The box represents the interquartile range, the whiskers indicate the lower and upper quartiles, the horizontal line within the box indicates the median and the “ + ” shows the mean. **B** Geometric mean ratios of quantitative ^68^Ga-FAPI-PET-parameters and ^18^F-FDG-PET-parameters of all metastatic lesions

**Fig. 2:**
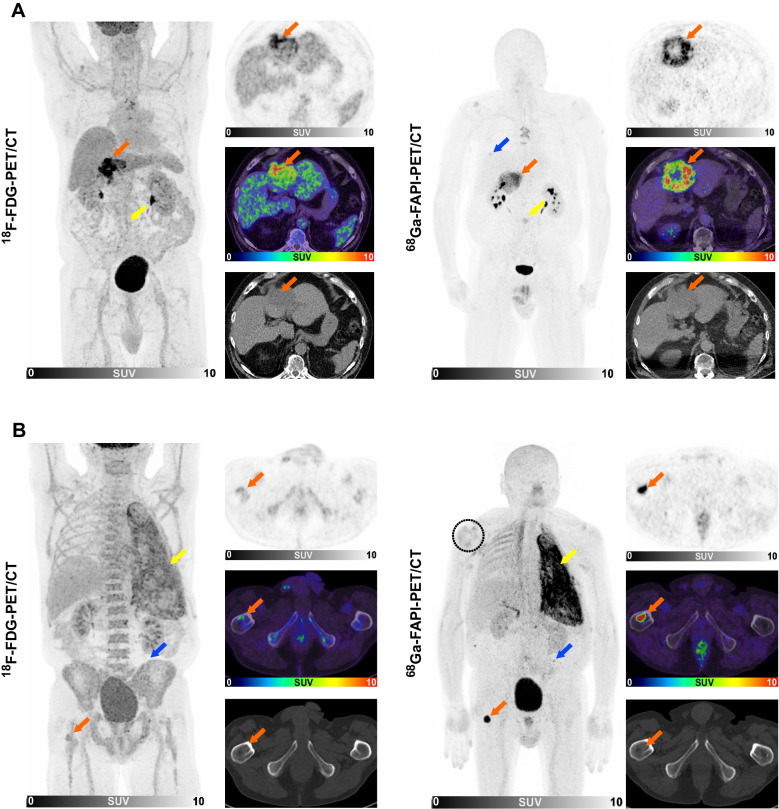
Representative images of ^18^Fluor-fluorodeoxyglucose- (^18^F-FDG) and ^68^Gallium-labelled-fibroblast activation protein inhibitor- (FAPI) positron emission tomography with computed tomography (^68^Ga-FAPI-PET/CT) in 2 patients with oligo-metastatic extra-cervical CUP. **A** 76 years old patient with an intrahepatic metastasis (red arrow) detectable in both, ^18^F-FDG- and ^68^Ga-FAPI-PET/CT, but more clearly delineated in the ^68^Ga-FAPI-PET/CT scan due to higher contrast with the liver background. ^68^Ga-FAPI-PET/CT shows additionally a lesion above the dome of the liver consistent with peritoneal carcinomatosis (blue arrow) and a bone lesion in the vertebra (yellow arrow), which cannot be clearly distinguished in ^18^F-FDG-PET/CT. **B** 77 years old patient with pleural carcinomatosis on the left side (yellow arrow) and a bone metastasis in the proximal right femur (red arrow) which is better distinguishable in ^68^Ga-FAPI- compared to ^18^F-FDG-PET/CT due to better tumor-to-tissue contrast. In the left ilium, ^68^Ga-FAPI-PET/CT also shows a second bone metastasis (blue arrow), which cannot be clearly delineated in ^18^F-FDG-PET/CT due to the bone marrow activation (displayed in Fig. 3). As a secondary finding, the right shoulder joint appears more intensely in the ^68^Ga-FAPI-PET/CT scan (dashed black circle)

Tracer-uptake was examined in 21 metastatic lesions of 9 patients. In 4 patients, who previously had extirpation of lymph node (LN) metastases, neither FDG- nor FAPI-uptake was observed. Mean SUVmax was slightly higher in ^68^Ga-FAPI- compared to ^18^F-FDG-PET/CT (FAPI: 7.36 ± 6.53; FDG: 6.15 ± 4.39) while mean SUVmean was similar (FAPI: 4.12 ± 3.04; FDG 4.04 ± 2.83). Mean TBRmax were significantly higher in ^68^Ga-FAPI-PET/CT (6.67 ± 6.43) compared to ^18^F-FDG-PET/CT (3.83 ± 3.37) and mean TBRmean (FAPI: 3.62 ± 2.88; FDG: 2.51 ± 2.29) showed a similar tendency. Mean TTRmax/mean was also higher in ^68^Ga-FAPI- (10.90 ±10.64/6.40± 6.85) compared to ^18^F-FDG-PET/CT (7.50 ± 6.73/4.78/4.50) (Fig. 1). Geometric mean ratios (FAPI/FDG), 95%-confidence intervals and p-values for all parameters are provided in supplemental Table 2. An exploratory subanalysis of tracer-uptake in 6 treated patients (13 lesions) and 3 treatment-naïve patients (8 lesions) showed markedly higher mean TBRmax/mean and TTBmax/mean in treatment-naïve patients for ^18^F-FDG, while mean SUVmax/mean was similar in both treatment-naïve and treated patients. Interestingly, for ^68^Ga-FAPI, mean SUVmean, TBRmean and TTBmax/mean were higher in treated patients, while mean SUVmax and TBRmax were higher in treatment-naïve patients. A detailed overview of uptake in these subgroups is provided in supplemental Table 3. Besides, tracer-uptake in joints was higher for ^68^Ga-FAPI compared to ^18^F-FDG in both treated and treatment-naïve patients. Additionally, in one patient, fibrotic lung changes presented more intense in ^68^Ga-FAPI- than ^18^F-FDG-PET/CT. Figure 2 shows two representative cases demonstrating the differential signal behavior of both tracers in metastatic sites in two patients with oligometastatic CUP.

### Head-to-head comparison of FDG- and FAPI-uptake

**Fig. 3:**
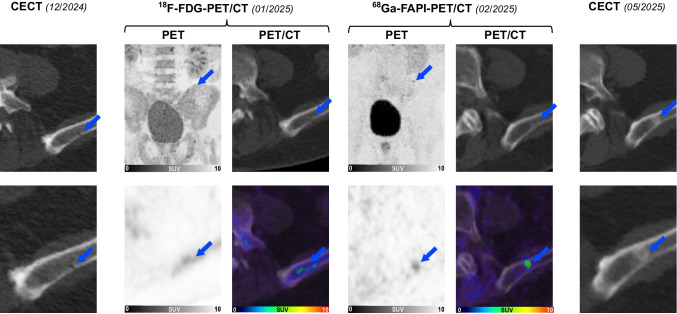
Exemplary case of a bone lesion which was detectable in ^68^Ga-FAPI- but not in ^18^F-FDG-PET (blue arrow). In a previous contrast enhanced CT (CECT) a small osteolysis is already visible in the left ilium (same patient as in 2B). 6 month later, after chemotherapy, blastic demarcation of an initially lytic lesion is evident in a CECT at the site where the ^68^Ga-FAPI-PET/CT previously showed circular enhancement, consistent with response to treatment of an osseous metastasis

6/21 metastatic lesions, one in the spleen, two in the bone, two in the liver and one lesion consistent with peritoneal carcinomatosis which bordered the dome of the liver, were only distinguishable in the ^68^Ga-FAPI-PET/CT. Three of these lesions were observed in the patients which were examined 26 days after ^18^F-FDG-PET/CT. Nevertheless, 2/3 lesions were already definable morphologically in previous diagnostic and low dose CT of ^18^F-FDG-PET/CT scan. Given this different detection rate, a sub-analysis of different metastatic sites was performed. In 9 lymph nodes, mean SUVmax/mean in ^68^Ga-FAPI- and ^18^F-FDG-PET/CT were similar, while mean TBRmax/mean was slightly higher in ^68^Ga-FAPI-PET/CT. Mean TTRmax/mean were comparable for both tracers with a slight advantage for ^18^F-FDG-PET/CT (FAPI: 8.18 ± 8.47/4.99 ± 4.87; FDG: 8.90 ± 4.85/5.90 ± 3.77). In 12 other metastatic sites (3 liver metastases, 1 peritoneal carcinomatosis which bordered the edge of the liver, 4 bone metastases, 1 splenic metastasis, 2 lung metastases and one pleural carcinomatosis) mean SUVmax/mean were comparable while TBRmax (FAPI: 6.93 ± 6.94; FDG: 3.53 ± 4.16) and TTRmax (FAPI: 12.94 ± 11.96; FDG: 6.46 ± 7.91) were significantly higher in ^68^Ga-FAPI-PET/CT. TBRmean (FAPI: 3.41 ± 2.58; FDG: 2.32 ± 2.87) and TTRmean (FAPI: 7.46 ± 8.08; FDG: 3.95 ± 5.17) were not significantly higher in ^68^Ga-FAPI-PET/CT (supplemental Fig. 1). Detailed geometric mean ratios (FAPI/FDG), 95%-confidence intervals and p-values for lymph node metastases and metastases other than lymph nodes are reported in supplemental Table 4 and 5. Mean SUVmax/mean and corresponding TBR and TTR values of all metastatic sites are given in Table 6. Figure 3 shows an exemplary case of a patient with a bone lesion which was detectable earlier in a diagnostic CT-scan, then was only visible in FAPI-PET/CT and later clearly unmasked as a bone metastasis.

## Discussion

The present study addresses a gap in current CUP research providing an intraindividual comparison of ^68^Ga-FAPI- and ^18^F-FDG-PET/CT in patients with extra-cervical CUP. Our findings indicate a clear advantage of ^68^Ga-FAPI-PET/CT with respect to TBRmax for all metastatic lesions and TTRmax in organ metastases leading to detection of additional metastatic lesions not detected by ^18^F-FDG-PET/CT. With respect to detection of lymph node metastases, ^68^Ga-FAPI- and ^18^F-FDG-PET/CT showed mostly equivocal detection rates and signal intensities. Additionally, we observed higher ^68^Ga-FAPI-uptake in joints and in fibrotic lung changes.

When interpreted in the context of previously published HNCUP studies, our data suggest a more nuanced role for ^68^Ga-FAPI-PET/CT for CUP assessment compared to earlier reports that observed superiority of ^68^Ga-FAPI- over ^18^F-FDG-PET/CT for detection of primary tumors and metastases in all organ systems. These studies have suggested that ^68^Ga-FAPI- may outperform ^18^F-FDG-PET/CT in CUP, particularly in HNCUP [15, 17, 21]. Gu et al. compared prospectively ^68^Ga-FAPI- with ^18^F-FDG-PET/CT in patients with HNCUP and reported higher detection rates of suspected primary tumors in ^68^Ga-FAPI-PET/CT outperforming the diagnostic accuracy of conventional imaging and ^18^F-FDG-PET/CT and leading to changes in oncologic treatment whereas ^18^F-FDG-PET/CT detected all metastatic lesions (lymph nodes and bone) with significantly higher SUVmax [15]. Shu et al. conducted a single-center prospective study in 44 patients with CUP and negative or equivocal findings in ^18^F-FDG-PET/CT. They analyzed the detection rates of primary tumors and lymph node metastases using ^68^Ga-FAPI-PET/CT demonstrating a slightly, non-significant difference in tracer-uptake (^68^Ga-FAPI > ^18^F-FDG) and a significantly higher TBR in ^68^Ga-FAPI- compared to ^18^F-FDG-PET/CT [17]. Serfling et al. evaluated both tracers in carcinomas in the Waldeyer´s tonsillar ring and showed comparable detection rates of primary tumors for both tracers while SUVmax and TBRmax of ^68^Ga-FAPI was much higher leading to an easier visual detection of tumor tissue. In contrast, the detection rate of lymph node metastases was markedly lower in ^68^Ga-FAPI-PET/CT [16]. Our results partially align with these observations. We similarly observed slightly higher SUV and a significantly higher TBRmax for ^68^Ga-FAPI- compared to ^18^F-FDG-PET/CT. Interestingly differences between both tracers were more pronounced for SUV/TBRmax- than for SUV/TBRmean-values. This may be explained by the heterogeneous intratumoral distribution of intensively positive cancer associated fibroblasts (CAFs), as observed in several studies of our group [28–31], which are reflected more by SUVmax than by SUVmean in ^68^Ga-FAPI-PET/CT. Furthermore, a previous analysis of texture parameters of PET data supports this hypothesis. In benign and malignant lung lesions, the combination of two texture parameters yielded higher diagnostic specificity than using the semi-quantitative parameters SUVmax/mean, a. finding which can be attributed to the heterogeneous distribution of CAFs in tumors [32]. However, this difference applied to organ metastatic lesions only, whereas no consistent improvement in primary tumor detection and equivocal results for lymph node metastases detection were observed across the cohort. In addition to the smaller number of patients compared to previous studies, this may in part be due to the fact that many patients had already received systemic treatment, including targeted therapies, by the time sequential imaging was performed. Prior therapy may have altered tracer-uptake patterns, particularly for ^18^F-FDG, which is sensitive to metabolic suppression following effective systemic treatment. In contrast, ^68^Ga-FAPI uptake may persist in fibrotic or reactive stromal components.

As in ^68^Ga-FAPI-PET/CT more metastatic lesions were detected compared to ^18^F-FDG-PET/CT, an additional analysis of TBR and TTR for organ metastases (bones, liver and lung) revealed a higher contrast for bone and liver metastases for ^68^Ga-FAPI, while for lymph node metastases the diagnostic performance of both tracers was comparable with a slight advantage for ^18^F-FDG. In lung metastases, ^68^Ga-FAPI showed a slightly higher contrast than ^18^F-FDG with generally the same detection rate. These findings are partially in line with previous study results [33–35]. Wu et al., for example, compared ^68^Ga-FAPI- and ^18^F-FDG-PET/CT with respect to the detection rate of bone metastases in 25 patients with lung cancer. They described a higher detection rate of metastases on lesion level as well as a higher image contrast and SUVmax in ^68^Ga-FAPI- compared to ^18^F-FDG-PET/CT [33]. While the superiority of ^68^Ga-FAPI-PET/CT in detecting bone, liver or lung metastases has been consistently reported, the results regarding the detection of lymph node metastases are mixed [16, 17, 34, 35]. On the one hand Shu et al. and Wang et al., who evaluated the performance of ^68^Ga-FAPI- and ^18^F-FDG-PET/CT regarding the detection of multiple metastatic regions in patients with advanced lung cancer, described a better performance of ^68^Ga-FAPI- compared to ^18^F-FDG-PET/CT including for lymph node metastases [17, 35]. On the other hand, Serfling et al. as well as Liu et al., who analyzed the performance of both tracers with regard to the detection of lymph node metastases of abdominal and pelvic malignancies, showed a lower detection rate of lymph node than of distant metastases in ^68^Ga-FAPI- compared to ^18^F-FDG-PET/CT [16, 34] which is in line with our results. Therefore, prospective studies are needed to gain a deeper understanding of the cases in which ^68^Ga-FAPI- is the more beneficial option for patients with lymph node metastases compared to ^18^F-FDG-PET/CT.

Given the different added value of ^18^F-FDG and ^68^Ga-FAPI for detecting metastases, the idea of a combined application of both tracers comes into focus. Roth et al. developed a dual-tracer PET/CT-protocol where patients with HN-tumors and esophageal cancer were first examined after injection of ^18^F-FDG followed by an additional scan after the application of ^68^Ga-FAPI [36]. As also demonstrated by further studies [37–39], the combined ^18^F-FDG/^68^Ga-FAPI-46-PET/CT showed higher TBR and lead to the detection of more suspicious lesions than ^18^F-FDG-PET/CT alone, partly due to increased uptake in lymph nodes that had previously shown ambiguous ^18^F-FDG-uptake. On the other hand, more suspicious lesions were detected in the liver following FAPI administration [36]. Despite increased time commitment, this approach of “one-stop” ^18^F-FDG/^68^Ga-FAPI-PET/CT offers diagnostic advantages. Combined PET diagnostics could enable a faster initiation of therapy, as unclear ^18^F-FDG-PET/CT findings can be immediately supplemented with ^68^Ga-FAPI-PET/CT results. The resulting more precise assessment of tumor burden could also optimize the choice between local treatment options in single-site situations or systemic treatment options in the oligometastatic stage, particularly when combined imaging leads to upstaging from single-site to oligometastatic disease. Combined ^18^F-FDG/^68^Ga-FAPI-PET/CT, particularly with contrast-enhanced CT, may offer a promising path towards faster, more accurate staging. However, more studies with larger cohorts are required to confirm its clinical benefits. To optimize efficiency and reduce patients´ radiation exposure, future protocols should investigate the simultaneous tracer administration, reduced doses and early PET acquisition protocols, exemplary after 10 min. Previous studies have shown similar detection rates in early and late ^68^Ga-FAPI-PET/CT as well as in dual-tracer PET/CT with reduced administered activity [39–41].

As a secondary finding, we observed increased ^68^Ga-FAPI-uptake in benign or non-malignant conditions such as joints and fibrotic lung tissue. These incidental findings are in line with current literature, which shows, for example, FAPI-uptake unrelated to degenerative changes [42, 43]. In particular, uptake in joints may be relevant findings for the clinical management of patients receiving immune checkpoint blockade therapy, as arthralgia and arthritis account for a significant proportion of side effects in these patients, as has been reported on several occasions [44–47].

### Limitations

Some limitations of our analysis must be taken into account. Mainly, the small number of patients should be mentioned which limits the number of analyzed metastases on the one hand and the statistical power on the other hand. In addition, almost half of the patients received systemic therapy including targeted therapy prior to PET-imaging, which could have made it difficult to detect primary tumors or metastases either in cases of good response or due to possible changes in tumor tissue. In this context it should be taken into account, that the exploratory subgroup analysis of treated and treatment-naïve patients is particularly limited by the small number and unbalanced distribution of lesions per group. This makes the analysis prone to outliers, as reflected by the high standard deviations. Therefore, this descriptive overview serves primarily to draw attention to the potential effects of therapies on the uptake behavior of both tracers with regard to, for example, stromal remodeling and tumor metabolic activity. These effects should be investigated in future larger-scale studies. Finally, regarding the interpretation of lesions detected on ^68^Ga-FAPI-PET/CT but not on ^18^F-FDG-PET/CT, it should be noted that due to the small number of patients, the two outliers in the time interval between both examinations could carry the risk that new lesions have developed in the interim. However, since five out of the six lesions that were detected only in ^68^Ga-FAPI-PET/CT were already morphologically definable in previous ^18^F-FDG-PET/CT, the risk of time-related disease progression in our cohort and thus of overestimating the potential of ^68^Ga-FAPI-PET/CT is considered low.

## Conclusion

Compared to existing literature in the field of CUP, especially HNCUP, our study addresses a gap in current CUP research by focusing on extra-cervical, single-site and oligometastatic CUPs representing a heterogeneous CUP population encompassing multiple underlying tumor entities with variable stromal composition. In comparison to ^18^F-FDG-, ^68^Ga-FAPI-PET/CT demonstrated a comparable detection rate with regard to lymph node metastases, but superiority in the detection of organ metastases, especially in the liver and bones. Taken together, our findings suggest that ^68^Ga-FAPI- appears favourable compared to ^18^F-FDG-PET/CT in extra-cervical, single-site and oligometastatic CUP, particularly in patients with suspected distant metastases other than lymph nodes, especially liver and bone. With regard to the small number of lesions, the heterogeneity of histological subgroups and different treatment status, larger prospective studies are needed to confirm our findings. In addition, as we did not detect any primary tumors in our cohort, further investigations are needed to compare both tracers under this aspect.

## Supplementary Information

Below is the link to the electronic supplementary material.

Supplementary file1 (DOCX 278 KB)

## Data Availability

The data that support the findings of this study are not openly available due to reasons of sensitivity and are available from the corresponding author upon reasonable request. Data are located in controlled access data storage at University Medical Center Mainz.
