# Supplementary material for: 68Ga-FAPI-PET/CT in extra-cervical CUP: Head-to-Head Comparison of 68Ga-FAPI-46 with 18F-FDG in 13 patients
Source: Eur J Nucl Med Mol Imaging. 2026 May 22;53(10):5833–43. doi: 10.1007/s00259-026-07916-0 (PMC13421285; doi:10.1007/s00259-026-07916-0)
Supplement: Supplementary file 1 — Supplementary file1 (DOCX 278 KB) [file 259_2026_7916_MOESM1_ESM.docx]

**Supplemental material**

**^68^Ga-FAPI-PET/CT in extra-cervical CUP: Head-to-Head Comparison of ^68^Ga-FAPI-46 with ^18^F-FDG in 13 patients**

Anna-Maria Spektor^1^, Sergio Armando Zapata Bonilla^2^, Philipp Mildenberger^3^, Anton Kilburg^4^, Daniela Driess^1^, Erik Winter^5^, Michael Kloth^6^, Mathias Schreckenberger^1^, Tobias Bäuerle^4^, Thomas Kindler^2,7,8,9^, Uwe Haberkorn^10,11,12^, Maria Pouyiourou^13,14^, Alwin Krämer^13,14^, Tilmann Bochtler^13,15^, Manuel Röhrich^1^

**Affiliations:**

1. Department of Nuclear Medicine, University Medical Center, Johannes Gutenberg University Mainz, Mainz, Germany.
2. University Cancer Center, University Medical Center, Johannes Gutenberg University Mainz, Mainz, Germany.
3. Institute of Medical Biometry, Epidemiology and Informatics, Johannes Gutenberg University Mainz, Mainz, Germany.
4. Department of Diagnostic and Interventional Radiology, University Medical Center, Johannes Gutenberg-University Mainz, Mainz, Germany.
5. Department of Nuclear Medicine, University Hospital Heidelberg, Heidelberg, Germany.
6. Department of Pathology, University Medical Center, Johannes Gutenberg-University Mainz, Mainz, Germany.
7. 3rd Medical Department, University Medical Center, Johannes Gutenberg University Mainz, Mainz, Germany.
8. TRON-Translational Oncology, University Medical Center, Johannes Gutenberg University Mainz, Mainz, Germany.
9. German Cancer Consortium (DKTK), partner site Frankfurt/Mainz, a partnership between DKFZ and University Medical Center Mainz, Mainz, Germany.
10. Department of Nuclear Medicine, University Hospital Heidelberg, Heidelberg, Germany.
11. Translational Lung Research Center Heidelberg (TLRC), Member of the German Center for Lung Research DZL, Heidelberg, Germany.
12. Clinical Cooperation Unit Nuclear Medicine, German Cancer Research Center (DKFZ), Heidelberg, Germany.
13. Clinical Cooperation Unit Molecular Hematology/Oncology, German Cancer Research Center (DKFZ) and Department of Internal Medicine V, University of Heidelberg, Heidelberg, Germany
14. Department of Internal Medicine V, University of Heidelberg, Heidelberg, Germany.
15. Department of Medical Oncology, Heidelberg University Hospital, Medical Faculty Heidelberg, Heidelberg, Germany.

**Corresponding author:**

Sergio Armando Zapata Bonilla

Langenbeckstr. 1

55131 Mainz, Germany

Telephone: +49 06131 17 5717

Email: [Sergio.ZapataBonilla@unimedizin-mainz.de](mailto:Sergio.ZapataBonilla@unimedizin-mainz.de)

**Supplemental Table 1**: Comprehensive IHC and molecular profiling

| **Patient** | **Histology** | **Key IHC Markers**^†^ | **NGS Performed** | **Key Mutations**^‡^ | **Targeted Therapy received prior PET/CT** |
| --- | --- | --- | --- | --- | --- |
| 1 | Urothelial | CK7+,p40+,PAX8+ | Yes | BRAF-V600E PIK3Ca E542L | no |
| 2 | Adenocarcinoma | CK7+, Desmi -, Thyreoglobulin-, CD10+, Ca125+, CK20-, GATA3-, Vimentin-, ER-, PR-, TTF1-, Ca19-9-, CDX2-, PD-L1 (IPS 3%, CPS 3, TPS 0%) | Yes | No actionable mutations | Ipilimumab/ Nivolumab |
| 3 | Squamous | CK 5/6+, CK7-, p16- | No |  | no |
| 4 | Squamous | p63+,p40-/+,p16-,PD-L1 5% HPV-DNA-; TMB 47 | Yes | No actionable mutations | Pembrolizumab |
| 5 | Adenocarcinoma | CK7+, CK20-, ER-, PR-, Her2neu 1+, GATA3+, CK19+, CDX2-, TTF1-, Thyreoglobulin-, WT1-, Chromogranin-, Mammaglobin-, AE1/3+ | No |  | Pembrolizumab |
| 6 | Squamous | CK7+,CK20+/-, GATA3+, TTF1-, ER-, PR-, PAX8-, p63+ | No |  | no |
| 7 | Adenocarcinoma | AE1/3+, TTF1+, Napsin+, GATA3-, p40-, CD56-, PD-L1 TPS 0% IC 0 CPS 0 | Yes | KRAS-G12V, STK11 mut, KEAP1mut | no |
| 8 | Squamous | AE 1/3+, PIT1-, TPIT-, GATA3-, p63+, CK5/6+, CK7-, CK20-, TG-, Napsin- | No |  | no |
| 9 | Adenocarcinoma | CK7+, CK20-, PAX8 weak, CDX2-, ER- | No | CHEK2mut, HRD-Score high (51) | no |
| 10 | Squamous | CK7+, CK5/14(+), p63+, CK5/6-, Chromogranin A-, HMB45-m TTF1- | No |  | Pembrolizumab |
| 11 | Adenocarcinoma | CK7+, GATA3+, HMB45-, Melan A, SOX10-, CD117-, ER-, Mammaglobin-, PSMA-, PAP-, PSA-, Adipophilin-, TTF-1-, p40-, DOG1-, NTRK-, Her2/neu 1+, p16 -, PD-L1 TPS 0%, CPS 5, IC 5% | Yes | BRCA1 c.4183C>T/p.Q1395*  BRCA2 (V) c.1597A>G/p.T533A  PTEN c.243del/p.F81Lfs*18  TP53 c.783-1G>T/p.X261_splice | no |
| 12 | Adenocarcinoma | CK7+, CK20-, SATB2-, TTF-1-, NapsinA-, Calretin-, WT1- | Yes | STK11-R297K | no |
| 13 | Sarcomatoid | AE1/3+, BEP4-, S100-, CD45-, CK7+, SMARCA4+, TTF1-, Snaptophysin-, CK20-, CDX2-, Ca19-9-, Calretin -, AR-, PD-L1: TPS:95%, CPS:100, IC:2%, TAP:100% | Yes | No actionable mutations | No |
| Abbreviations: ^†^PD-L1 and p16 status were reported in selected cases only and were included in the IHC profile where available.  ^‡^ “No actionable mutations” includes mutations detected without therapeutic relevance | | | | | |

**Supplemental Table 2:** Ratio FAPI/FDG for all metastatic sites

| **Variable** | **Ratio** | **95%-CI** | **p-value** | **p-value_adj** |
| --- | --- | --- | --- | --- |
| SUVmax | 1.053 | (0.78 - 1.442) | 0.3710 | 0.429 |
| SUVmean | 0.976 | (0.714 - 1.366) | 0.4290 | 0.429 |
| TBRmax | 1.541 | (1.052 - 2.254) | 0.0120 | 0.072 |
| TBRmean | 1.443 | (0.985 - 2.121) | 0.0310 | 0.093 |
| TTRmax | 1.347 | (0.648 - 2.863) | 0.2215 | 0.354 |
| TTRmean | 1.297 | (0.648 - 2.669) | 0.2360 | 0.354 |
| SUVmax/mean: maximum/mean standardized uptake values; TBR: tumor-to-background ratio; TTR: tumor-to-tissue ratio; CI: Confidence interval; p-value: unadjusted p-value; p-value_adj: p-values adjusted using the Bonferroni-Holm method | | | | |

**Supplemental table 3: ^18^F-FDG- and ^68^Ga-FAPI-46-uptake in treated and untreated patients**

| **Patients (n)** | **Lesions**  **(n)** | **^18^F-FDG-PET/CT** | | | **^68^Ga-FAPI-46-PET/CT** | | |
| --- | --- | --- | --- | --- | --- | --- | --- |
|  |  | *SUVmax/*  *mean ±SD* | *TBRmax/*  *mean ±SD* | *TTBmax/*  *mean ±SD* | *SUVmax/*  *mean ±SD* | *TBRmax/*  *mean±SD* | *TTBmax/*  *mean±SD* |
| **Treatment-naïve (3)** | 8* | 6.11±4.97/  3.89±3.53 | 5.29±4.79/  3.40±3.38 | 10.86±7.51/  6.77±5.39 | 8.59±9.32/  3.49±3.53 | 8.41±9.30/  3.41±3.81 | 10.46±9.62/  4.61±5.07 |
| **Treated**  **(6)** | 13** | 6.18±4.21/  4.13±2.47 | 2.93±1.78/ 1.97±1.09 | 5.43±5.52/  3.56±3.55 | 6.61±4.33/  4.51±2.78 | 5.60±3.89/  3.76±2.31 | 11.17±11.60/  7.51±7.73 |
| Abbreviations: n: number; PET/CT: positron emission tomography with computed tomography; ^18^F-FDG: ^18^Fluor‑fluorodeoxyglucose; ^68^Ga-FAPI: ^68^Gallium-fibroblast activation protein inhibitor; SUVmax/mean: maximum/mean standardized uptake values; TBR: tumor-to-background ratio; TTR: tumor-to-tissue ratio; SD: standard deviation.  * 5 x lymph nodes, 1 x bone, 2 x lungs.  **4 x lymph nodes, 3 x bones, 3 x liver, 1 x spleen, 1 x pleura, 1 x peritoneal carcinomatosis bordering the edge of the liver. | | | | | | | |

**Supplemental Table 4:** Ratio FAPI/FDG for 9 lymph node metastases

| **Variable** | **Ratio** | **95%-CI** | **p-value** | **p-value_adj** |
| --- | --- | --- | --- | --- |
| SUVmax | 1.027 | (0.516 - 2.02) | 0.4635 | 0.4635 |
| SUVmean | 1.066 | (0.622 - 1.832) | 0.4315 | 0.4635 |
| TBRmax | 1.253 | (0.589 - 2.707) | 0.3090 | 0.4635 |
| TBRmean | 1.302 | (0.678 - 2.458) | 0.2245 | 0.4635 |
| TTRmax | 0.690 | (0.255 - 1.864) | 0.2365 | 0.4635 |
| TTRmean | 0.714 | (0.312 - 1.614) | 0.2265 | 0.4635 |
| SUVmax/mean: maximum/mean standardized uptake values; TBR: tumor-to-background ratio; TTR: tumor-to-tissue ratio; CI: Confidence interval; p-value: unadjusted p-value; p-value_adj: p-values adjusted using the Bonferroni-Holm method | | | | |

**Supplemental Table 5:** Ratio FAPI/FDG for 12 metastatic sites other than lymph nodes

| **Variable** | **Ratio** | **95%-CI** | **p-value** | **p-value_adj** |
| --- | --- | --- | --- | --- |
| SUVmax | 0.993 | (0.625 – 1.565) | 0.4730 | 0.4730 |
| SUVmean | 0.761 | (0.437 - 1.363) | 0.2135 | 0.3750 |
| TBRmax | 1.564 | (1.016 - 2.456) | 0.0295 | 0.0885 |
| TBRmean | 1.166 | (0.583 - 2.179) | 0.3330 | 0.3996 |
| TTRmax | 2.075 | (1.046 – 4.12) | 0.0205 | 0.0885 |
| TTRmean | 1.489 | (0.554 – 3.97) | 0.2500 | 0.3750 |
| SUVmax/mean: maximum/mean standardized uptake values; TBR: tumor-to-background ratio; TTR: tumor-to-tissue ratio; CI: Confidence interval; p-value: unadjusted p-value; p-value_adj: p-values adjusted using the Bonferroni-Holm method | | | | |

**Supplemental Table 6:** SUV, TBR and TTR of metastatic sites

| **Metastatic sites (n)** | **^18^F-FDG-PET/CT** | | | **^68^Ga-FAPI-46-PET/CT** | | |
| --- | --- | --- | --- | --- | --- | --- |
|  | *SUVmax/*  *mean (±SD)* | *TBRmax/*  *mean (±SD)* | *TTRmax/*  *mean (±SD)* | *SUVmax/*  *mean (±SD)* | *TBRmax/*  *mean (±SD)* | *TTRmax/*  *mean (±SD)* |
| **Lymph nodes (9)** | 6.05±2.95/ 3.97±1.99 | 4.23±2.05/ 2.77±1.27 | 8.90±4.85/ 5.90±3.37 | 7.04±5.51/  4.50±3.26 | 6.32±6.06/  3.90±3.39 | 8.18±8.47/ 4.99±4.87 |
| **Other than LN (12)** | 6.23±5.36/  4.09±3.42 | 3.53±4.16/  2.32±2.87 | 6.46±7.91/  3.95±5.17 | 7.60±7.44/  3.84±2.99 | 6.93±6.94/  3.41±2.58 | 12.94±11.96/  7.46±8.08 |
| **Bone (4)** | 7.50±6.06/ 5.26±4.21 | 5.44±6.93/ 3.81±4.82 | 8.17±12.25/ 5.67±8.54 | 11.81±10.30/ 4.64±3.38 | 10.39±9.55/ 3.79±1.65 | 19.65±14.85/  10.78±12.10 |
| **Liver (3) + peritoneum* (1)** | 7.02±7.32/ 4.63±4.12 | 2.84±2.58/ 1.88±1.42 | 2.38±2.32/ 1.58±1.29 | 4.74±3.07/ 3.12±1.75 | 6.38±6.36/ 4.07±3.67 | 6.95±5.09/ 4.53±2.87 |
| **Lung (2) + pleura (1)** | 4.73±2.77/ 2.50±1.78 | 2.65±1.06/ 1.36±0.68 | 11.39±4.56/ 5.82±2.86 | 6.88±8.55/ 4.01±4.97 | 4.34±5.06/ 2.47±2.99 | 14.94±14.50/  8.51±8.71 |
| **Spleen (1)** | 2.50/2.02 | 1.28/1.03 | 1.10/0.89 | 4.41/3.03 | 3.09/2.12 | 4.09/2.81 |
| Abbreviations: n: number of lesions; LN: lymph nodes; PET/CT: positron emission tomography with computed tomography; ^18^F-FDG: ^18^Fluor‑fluorodeoxyglucose; ^68^Ga-FAPI: ^68^Gallium-fibroblast activation protein inhibitor; SUVmax/mean: maximum/mean standardized uptake values; TBR: tumor-to-background ratio; TTR: tumor-to-tissue ratio; SD: standard deviation.  *: peritoneal carcinomatosis bordered the edge of the liver | | | | | | |

**Supplemental figure 1**


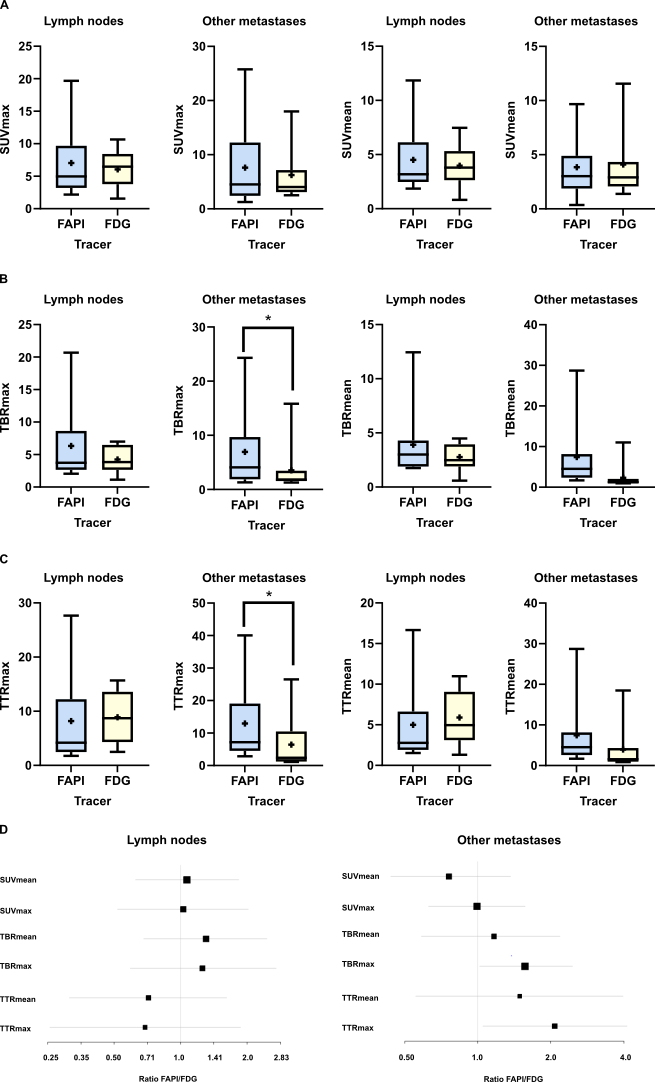


Box-Whisker-Plot of **A** maximum and mean standardized uptake values (SUVmax/mean), **B** tumor-to-background ratios (TBRmax/mean) and **C** tumor-to-tissue ratios (TTRmax/mean) of 9 lymph node metastases and 12 other metastatic lesions (4 bone, 3 liver, 1 peritoneal carcinomatosis bordering the liver dome, 2 lung, 1 pleura carcinomatosis, 1 splen) of 9 patients with extra-cervical CUP who were examined with positron emission tomography combined with computed tomography (PET/CT) with ^68^Gallium-labelled fibroblast activation protein inhibitor (^68^Ga-FAPI) and ^18^Fluor-labelled fluorodeoxyglucose (^18^F-FDG). Significant differences (*) were observed for TBRmax and TTRmax in metastatic lesions other than lymph nodes. The box represents the interquartile range, the whiskers indicate the lower and upper quartiles, the horizontal line within the box indicates the median and the “+” shows the mean. **D** Geometric mean ratios of quantitative ^68^Ga-FAPI-PET-parameters and ^18^F-FDG-PET-parameters of lymph node metastases and other metastases.

**Supplemental figure 2**


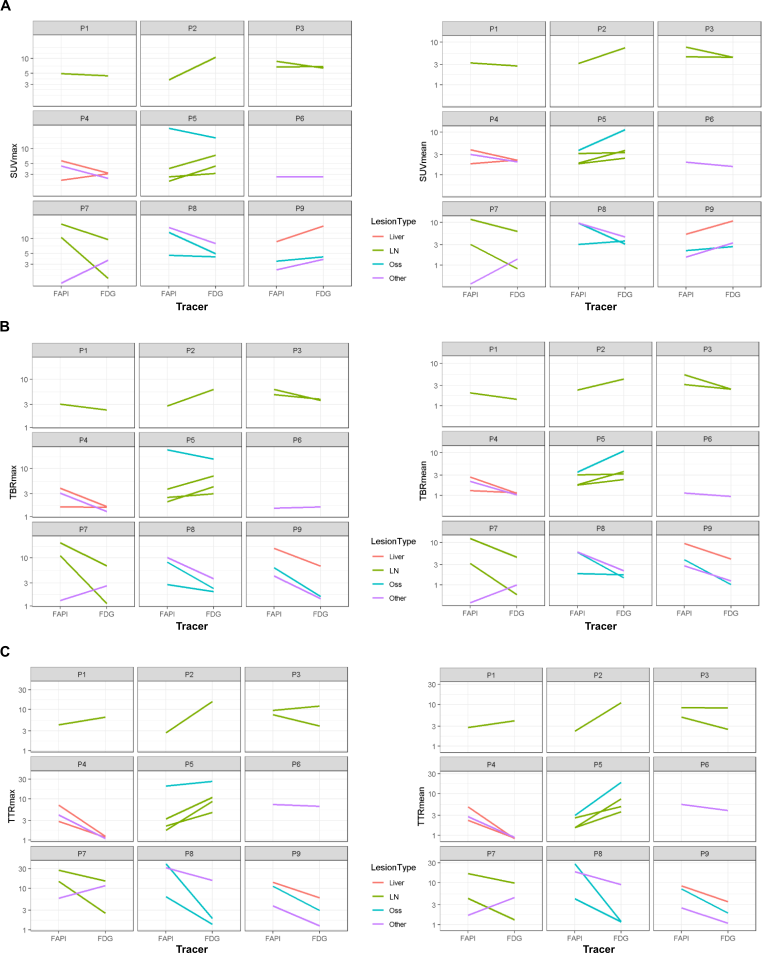


**A** Standardized uptake values (SUVmax/mean) **B** tumor-to-background ratio (TBRmax/mean) and **C** tumor-to-tissue ratio (TTRmax/mean) of metastatic lesions (liver and peritoneal carcinomatosis bordering the liver dome (liver, red), lymph nodes (LN, green), bones (Oss, blue), lung, pleural carcinomatosis, spleen (other, purple) across nine patients (P1-P9). Each line represents the two measurements for one lesion, ^68^Ga-FAPI (left) and ^18^F-FDG (right). An upward slope indicates higher tracer uptake for ^18^F-FDG compared to ^68^Ga-FAPI, while a downward slope indicates higher uptake for ^68^Ga-FAPI compared to ^18^F-FDG.
